# Bioinformatics Analysis of the *FREM1* Gene—Evolutionary Development of the IL-1R1 Co-Receptor, TILRR

**DOI:** 10.3390/biology1030484

**Published:** 2012-09-25

**Authors:** Richard C. Hudson, Caroline Gray, Endre Kiss-Toth, Timothy J. A. Chico, Eva E. Qwarnstrom

**Affiliations:** Department of Cardiovascular Sciences, Medical School, University of Sheffield, Beech Hill Road, Sheffield S10 2RX, UK; Email: ricardo.hudson@gmail.com (R.C.H.); caroline.gray@sheffield.ac.uk (C.G.); e.kiss-toth@sheffield.ac.uk (E.K.-T.); t.j.chico@sheffield.ac.uk (T.J.A.C.)

**Keywords:** TILRR, IL-1RI, co-receptor, FREM1, bioinformatics, evolutionary development, signal transduction, TIR activation, IL-1

## Abstract

The TLRs and IL-1 receptors have evolved to coordinate the innate immune response following pathogen invasion. Receptors and signalling intermediates of these systems are generally characterised by a high level of evolutionary conservation. The recently described IL-1R1 co-receptor TILRR is a transcriptional variant of the *FREM1* gene. Here we investigate whether innate co-receptor differences between teleosts and mammals extend to the expression of the TILRR isoform of *FREM1*. Bioinformatic and phylogenetic approaches were used to analyse the genome sequences of *FREM1* from eukaryotic organisms including 37 tetrapods and five teleost fish. The TILRR consensus peptide sequence was present in the *FREM1* gene of the tetrapods, but not in fish orthologs of *FREM1*, and neither *FREM1* nor *TILRR* were present in invertebrates. The *TILRR* gene appears to have arisen via incorporation of adjacent non-coding DNA with a contiguous exonic sequence after the teleost divergence. Comparing co-receptors in other systems, points to their origin during the same stages of evolution. Our results show that modern teleost fish do not possess the IL-1RI co-receptor TILRR, but that this is maintained in tetrapods as early as amphibians. Further, they are consistent with data showing that co-receptors are recent additions to these regulatory systems and suggest this may underlie differences in innate immune responses between mammals and fish.

## 1. Introduction

The innate immune system is generally well conserved throughout the animal kingdom with the same characteristic features of regulatory components present in species ranging from insects to mammals [1]. Activation is induced through members of the Toll-like and IL-1 receptor (TIR) family, characterized by the cytoplasmic TIR domain [2]. The high level of conservation of the intracellular domains of both Toll and IL-1R1 and of cytoplasmic regulatory components is consistent with a signaling system that is broadly conserved throughout evolution prior even to the divergence of plants [3] and animals [4]. However, it is increasingly recognized that mechanisms of ligand recognition and co-receptor association, a potent regulator of signal amplification at the level of the receptor complex, are less well conserved [5,6]. In evolutionary terms, such co-receptors appeared relatively late in the development of their respective signaling networks which they control.

Recent studies have revealed that fish, which have been shown to possess certain inflammatory receptors, may lack co-receptors found in more modern organisms, suggesting that signaling mechanisms in earlier species are functionally distinct and less refined. Thus, for example the zebrafish (*Danio rerio*) possesses two paralogs of TLR4, neither of which is stimulated by LPS, and lacks the co-receptors MD2 and CD14 [7,8]. Similarly, phylogenetic studies of the synteny of the syndecan genes in fish and tetrapods has revealed that while the four mammalian syndecan genes arose due to gene duplication, Syndecan 1 (an FGFR co-receptor) is absent from fish genomes probably as a result of deletion following this duplication event [9].

We recently identified the IL-1R co-receptor, TILRR (Toll-like/IL-1 receptor regulator), a 715 amino acid heparan sulfate glycoprotein encoded within the gene for the extracellular matrix protein FREM1 [10]. FREM1 has a distinct function in embryogenesis and development, and is ubiquitously expressed [11].

TILRR binds the cell membrane through a *C*-terminal lectin domain, associates with IL-1R1 and increases receptor expression and ligand binding. TILRR association potentiates recruitment of the MyD88 adapter and receptor signal amplification, and enhances activation of NF-κB and inflammatory genes [10]. We earlier confirmed expression and function of TILRR in mouse and human cells [10]. 

The current studies examine the presence of TILRR throughout evolution and demonstrate that TILRR is a transcriptional variant of *FREM1* whose transcriptional start site lies within the intronic sequence of *FREM1*. TILRR is lacking in early species such as teleosts and invertebrates, being first identifiable in amphibians. These findings highlight that although innate immunity is evolutionarily ancient, refinements to the system have continued to arise until more recently and that important differences exist between model organisms used to study inflammation.

## 2. Results and Discussion

In order to determine the evolutionary development of the IL-1RI co-receptor TILRR, we identified the TILRR isoform of *FREM1* within the genomes of multiple organisms and defined the source of the TILRR peptide sequence within the nucleotide sequence of the *FREM1* loci. Alignment of the peptide sequences of human TILRR and FREM1 show that they are identical from R17 of TILRR (R1481 of FREM1). This TILRR/FREM1 consensus region is encoded by exons 25 onwards in the human *FREM1* gene.

**Figure 1:**
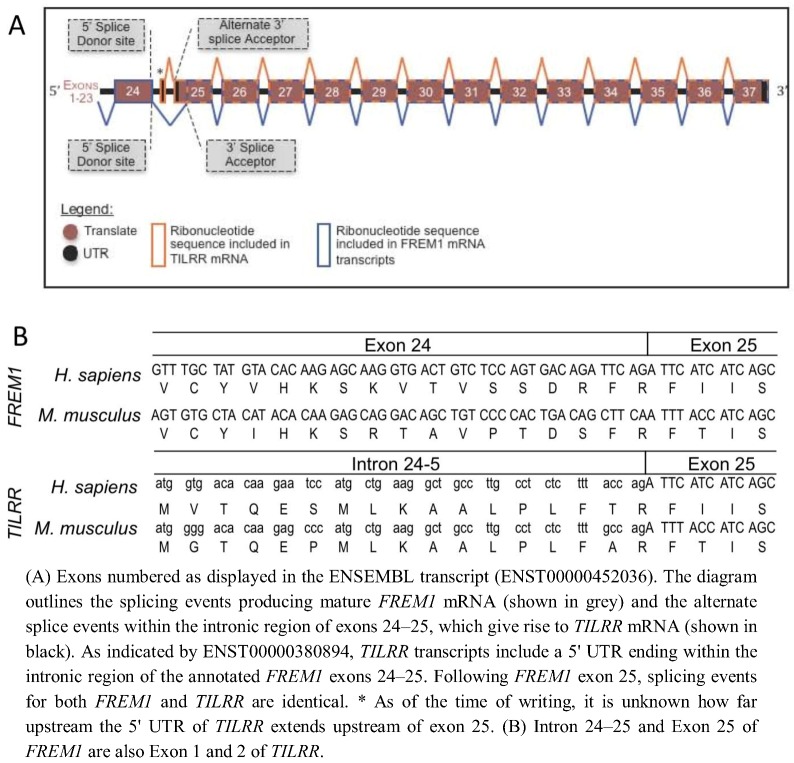
(**A**) Schematic diagram of Human and Mouse *FREM1* pre-mRNA. (**B**) DNA sequence and peptide translation of human and mouse *FREM1* in region of boundary between exons 24 and 25 (upper panel) and TILRR showing genomic sequence and translated peptide.

To predict the location within the Human *FREM1* locus where *TILRR* transcripts initiate, we analysed the Ensembl annotation of the 2,179 amino acid encoding Human *FREM1* gene (ENST00000452036). Studying the cDNA sequence of this transcript revealed R1481 to be encoded within a residue overlap splice site and due to ligation of the final two nucleotides of exon 24, and the first of exon 25 (Figure 1). A lack of homology between the 16 N-terminal TILRR residues and any other part of FREM1 suggested that no early *FREM1* coding exon is ligated to exon 25 to encode TILRR. Therefore, we reasoned that the *N*-terminus (translational start site) of TILRR must reside within a sequence of *FREM1* not incorporated into the processed *FREM1* mRNA. We hypothesized that the *N*-terminus of TILRR could be located by examining the annotation of the *FREM1* transcript, prior to the first exon common to both *TILRR* and *FREM1* (exon 25). Translation in all three reading frames of the intronic nucleotide sequence immediately preceding exon 25 produced the unique 16 TILRR *N*-terminal residues that we previously sequenced using MALDI-TOF [10]: MVTQESMLKAALPLFT followed by R17 and the remaining residues up to Q155. As the *N*-terminus of TILRR is produced by a series of consecutive nucleotides immediately prior to and in frame with the third nucleotide of the R1481 codon of FREM1, during RNA processing, *FREM1* mRNA transcripts arise when exon 24 is ligated to a 3' splice acceptor site prior to the third nucleotide of the R1481 codon, whereas exon 1 of *TILRR* commences within intron 23–24 and runs into exon 24 without the requirement for such an acceptor splice site (Figure 1B). This initiation of a novel “orphan” gene from a non-coding sequence is a recently described mechanism, which in many cases allows the organism to adapt to novel conditions [12,13,14].

Analysis of the Mouse *Frem1* locus (ENSMUSG00000059049) in the same manner as Human *FREM1,* revealed a similar splicing mechanism: the *N*-terminal 16 residues of Mouse TILRR (MGTQEPMLKAALPLFA, as we previously showed by peptide sequencing) [10] are encoded by an intronic nucleotide sequence upstream of exon 25 of the *Frem1* transcript. This is consistent with the suggestion that *TILRR* and *FREM1* mRNAs arise due to alternative transcriptional initiation of the *TILRR* mRNA within intron 24–25 of the *FREM1* gene.

Since analysis of both the Human *FREM1* and Mouse *Frem1* loci clearly identified the 5' *TILRR* coding sequence within the intron preceding exon 25, we reasoned that examining this region in other species would allow determination of whether each organism possesses an ortholog of *TILRR*.

**Figure 2:**
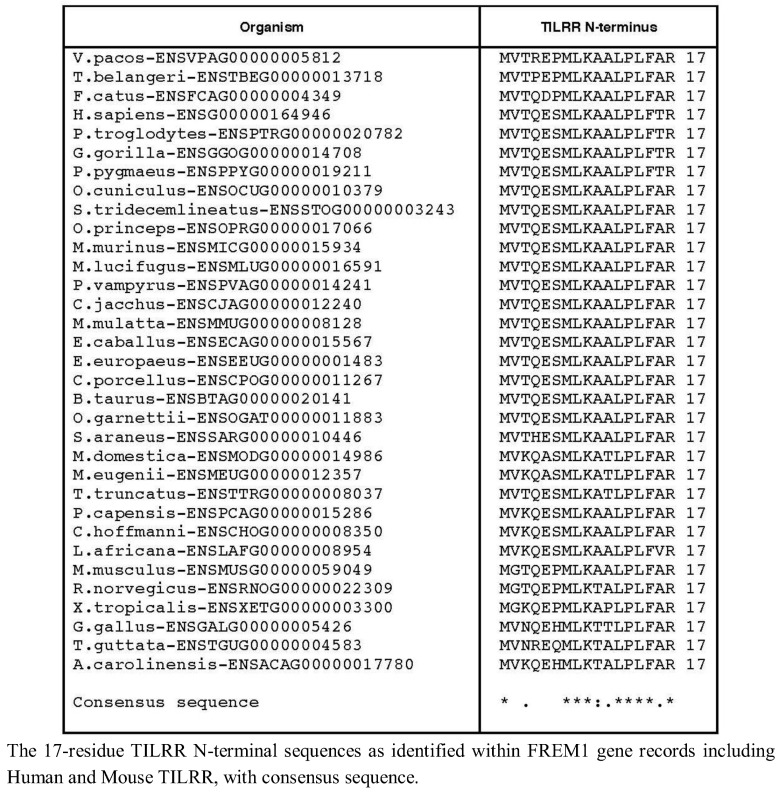
Comparison of TILRR *N*-terminal sequence.

We therefore extended our investigation to examining the genomes of 37 tetrapod organisms to identify the 5' end of the *TILRR* coding sequence within each Frem1 ortholog. We used the predicted *FREM1* cDNA transcript sequences to identify the region of each locus corresponding to the exon containing alternative 3' splice acceptor sites as in exon 25 of Human *FREM1*. The preceding nucleotides were translated in frame with the nucleotide sequence of the identified exon and the resulting peptide sequence aligned with the TILRR N-termini. For 33 tetrapod species, 16 consecutive amino acids could be produced in frame with R17, suggesting that a strongly conserved *TILRR* homolog exists in these organisms (Figure 2).

**Figure 3:**
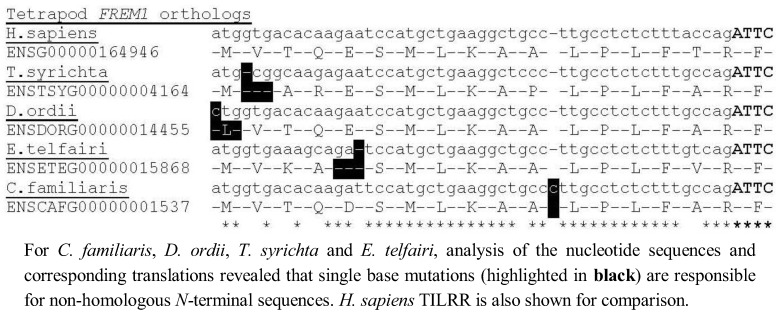
Identification of single base mutations responsible for non-homologous sequence in *C. familiaris*, *D. ordii*, *T. syrichta* and *E. telfairi*.

Although four tetrapod *FREM1* loci (*C. familiaris*, *D. ordii*, *T. syrichta* and *E. telfairi*) could not immediately be translated into the 5' TILRR residues, we found that all four possess a single nucleotide alteration compared to the consensus TILRR peptide sequence, but that with this exception the consensus *N*-terminal TILRR sequence was preserved, indicating these species are likely to possess the TILRR isoform of FREM1 (Figure 3). Alternatively, in these species *TILRR* may constitute a pseudogene, which produces a non-functional protein, although it is highly likely that all tetrapod homologues arose from a common ancestor. Future studies are needed to assess the significance of these mutations in signal amplification of the TIR domain.

**Figure 4:**
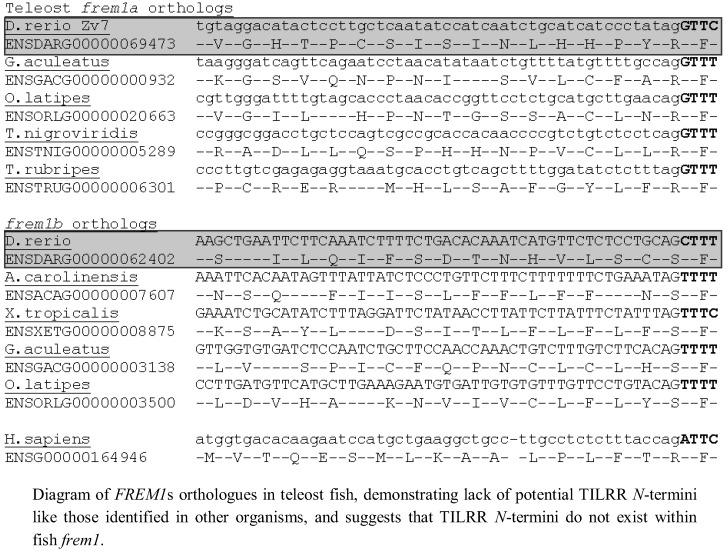
*FREM1* orthologs in fish lack the TILRR *N*-terminus.

Since we had identified TILRR orthologues in all tetrapod species studied, we next analysed teleost *FREM1* homologs using annotations of all identified teleost *FREM1* orthologs in the Ensembl database. All teleost species possessed at least one *FREM1* orthologue. However, in these organisms no such *FREM1* ortholog contained a conserved 5' coding sequence indicative of a *tilrr* transcript (Figure 4).

We next examined invertebrate species for orthologues of *FREM1*/*TILRR*. Using BLASTP, we were unable to locate orthologues of either the consensus regions shared between *Frem1* and *Tilrr* or the *Tilrr* 5' sequence in non-vertebrates (*Drosophila melanogaster*, *Ciona intestinalis, Caenorhabditis elegans*). We therefore concluded that invertebrates do not possess *FREM1*, nor a *TILRR* orthologue. Thus, *FREM1* arose after the evolution of vertebrates, but TILRR only becomes detectable after the divergence of the teleosts.

**Figure 5:**
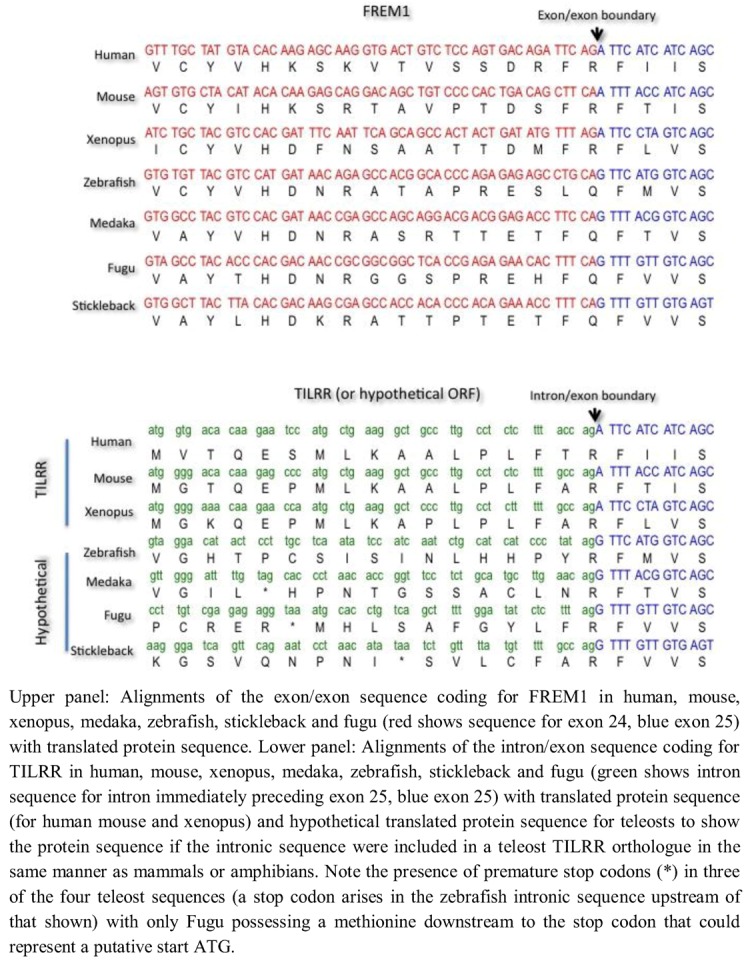
Comparative alignment of coding sequence of FREM1 and TILRR in mammals, amphibians and teleosts.

To investigate possible mechanisms for the absence of *TILRR* in teleosts, we examined the exon/exon boundary sequences of *FREM1* in human, mouse, xenopus and four teleosts (*Danio rerio* [zebrafish], *Oryzias latipes* [medaka], *Takifugu rubripes* [fugu] and *Gasterosteus aculeatus* [stickleback] and the corresponding intron/exon sequences that encode for TILRR in tetrapods but not in teleosts (Figure 5). We found that whereas the exonic and particularly translated sequence of *FREM1* were reasonably well conserved even between mammals and telosts, comparison of the intronic region encoding for TILRR in tetrapods reveals marked divergence. Although all introns end with the major spliceosomal AG consensus splice site, the actual intronic sequences diverged markedly between teleosts and mammals. Although N termini often vary greatly in length and sequence between homologues, in zebrafish, medaka and stickleback translation of the ORF of the contiguous intronic sequence preceding the shared *frem1* exon leads to a stop codon between the shared exon and the earliest possible methionine start codon (Figure 5). In Fugu there is a methionine in the N-terminal sequence that could represent a TILRR start codon, although there is no equivalent of this in the human sequence (Figure 5). Given the otherwise high conservation of the *frem1* gene between teleosts, it seems unlikely that Fugu possess a TILRR homologue when the other three teleosts do not. It seems likely that *TILRR* arose through a major alteration of intronic sequence, rather than a more subtle perturbation that gave rise to generation of novel intronic transcription binding sites.

**Figure 6:**
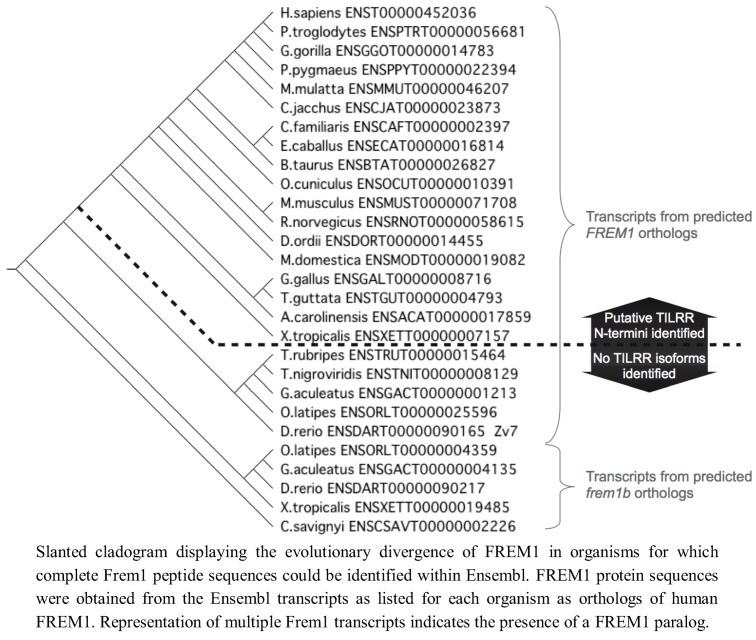
Evolutionary development of FREM1 and presence of putative TILRR sequence.

We therefore conclude that the *TILRR* isoform of *FREM1* is present only in tetrapod organisms, presenting two possibilities for the origin of *TILRR*. This may reflect that it originates from an ancestor common to both teleost and tetrapod organisms that arose after the invertebrates, and was lost in *FREM1* paralogs prior to the evolution of modern teleosts. Alternatively, (an explanation we favour) *TILRR* may have originated following the divergence of a common ancestor into the tetrapod lineage, hence its first detection within amphibian *FREM1* (Figure 6).

Either possibility suggests that, in contrast to a majority of IL-1RI complex components, which are present in primitive species such as *D. rerio* [7,9], *TILRR* is not involved in IL-1R1 controlled responses to pathogenic invasion in ancestral or modern day teleosts. 

The work in this study shows the maturation of the IL-1 receptor complex within the timeframe between the divergent evolution of teleost fish and tetrapod amphibians some 360–450 million years ago [15]. The conservation of *TILRR* within the genomes of tetrapod organisms likely represents refinement of IL-1 signaling over the course of vertebrate evolution, to allow increased sensitivity of system control through ligand concentration and receptor levels.

The finding that *TILRR* does not exist in any teleost studied suggests that distinct components of the vertebrate IL-1R1 complex may have evolved at different stages of the evolutionary tree, perhaps reflecting functions related to *TILRR* controlled environmental sensing and attachment. The lack of *TILRR* expression in primitive species, such as *D. rerio*, in addition to the absence of Syndecan 1 and the TLR4 co-receptors [8,9], also supports the notion that inflammatory signaling regulatory mechanisms in mammals are not all synonymous with that of lower vertebrates. Common features of the co-receptors of these systems are related to ligand/receptor interactions and receptor sensitivity to ligand, allowing for increasing variability and specificity over a range of ligand and receptor levels. Similarly, recently identified *TILRR* mutants demonstrate selective regulation of distinct cellular responses related to inflammation and cell survival, thus contributing refined control and increased specificity [16].

## 3. Experimental Section

### 3.1. Obtaining Predicted Frem1 Peptide Sequences

Genbank [17] was used as a source for TILRR and FREM1 peptide sequences. GeneIDs and NCBI [18] Reference sequences for the peptides used to compare with predicted transcripts are listed below (Table 1).

**Table 1:** Accession numbers of FREM1 sequences.

Organism	GeneID	Protein Name	NCBI Reference sequence
*H. sapiens*	158326	FREM1 isoform 2 (TILRR)	NP_001171175
		FREM1isoform 1 precursor	NP_659403
*M. musculus*	329872	FREM1 precursor	NP_808531
*D. rerio*	100216326	Frem1a	NP_001177237
	557221	Frem1b	NP_001131130

Ensembl was used to locate predicted *FREM1* gene loci, identified as orthologs of the Ensembl annotation of Human *FREM1* (ENSG00000164946) or of Zebrafish *frem1b* (ENSDARG00000062402) [19].

Predicted Ensembl *FREM1* ortholog transcripts are shown in Supplementary Figure 1.

Previous studies of *D. rerio* Frem1 used probes deduced by sequence analysis to analyse expression of the two orthologs, Frem1a and Frem1b, which we aligned to the predicted protein sequences as listed in Ensembl. By this method, we found that *D. rerio* Frem1a had a greater similarity to the ENSDART00000090165 transcript annotation in the Zv7 genome as compared to the more recent Zv8 genome annotation. Conversely, the *frem1b* transcript had perfect similarity to ENSDART00000090217 on Zv8. For these reasons we used the Zv7 annotation of ENSDART00000090165 as a basis for the exon structure of *frem1a*, and the Zv8 annotation of ENSDART00000090217 as a basis for the exon structure of *frem1b*.

### 3.2. Sequence Alignments

All peptide and nucleotide sequence alignments were performed using CLUSTALW and CLUSTALX. A slanted cladogram of FREM1 peptide sequences was constructed using the UPGMA algorithm in CLUSTALX displayed using TreeViewX.

## 4. Conclusions

Our data show that TILRR is a recent addition to the IL-1RI signaling system. These findings, and those of others looking at evolutionary development of regulatory pathways, are consistent with a role for co-receptors in modulating response control in higher organisms. This opens interesting possibilities for investigating development of regulatory intermediates and delineating mechanisms underlying the increased sensitivity and complexity characteristic of maturation of biological systems during evolution.

## Supplementary Files

Supplementary File 1 PDF-Document (PDF, 667 KB)

## Abbreviations

IL-1: interleukin 1
IL-1RI: interleukin 1 receptor type I
TILRR: Toll-like and Il-1 receptor regulator
NF-κB: nuclear factor kappa B
NCBI: National Center for Biotechnology Information
FREM-1: FRAS Related Extracellular Matrix gene 1
