# Supplementary material for: Bioinformatics Analysis of the FREM1 Gene—Evolutionary Development of the IL-1R1 Co-Receptor, TILRR
Source: Biology (Basel). 2012 Sep 25;1(3):484–94. doi: 10.3390/biology1030484 (PMC4009816; doi:10.3390/biology1030484)
Supplement: Supplementary File 1 — PDF-Document (PDF, 667 KB) [file biology-01-00484-s001.pdf]

Supporting Information File S1.

Table of organisms and sequences identified in the study, including information on the genes used and the genome versions studied.

Supporting Information File S1

Ensembl Frem1 orthologs

Organism	Ensembl Gene	Genome version	Sequence identified
**Vicugna pacos**	**ENSVPAG00000005812**	vicPac1	MVTREPMLKAALPLFAR
**Anolis carolinensis**	**ENSACAG00000017780**	AnoCar1.0	MVKQEHMLKTALPLFAR
**Dasypus novemcinctus**	**ENSDNOG00000007689**	dasNov2	corrupt DNA sequence
**Otolemur garnettii**	**ENSOGAG00000011871**	otoGar1	MVTQESMLKAALPLFAR
**Ciona savignyi**	**ENSCSAVG00000001283**	CSAV 2.0	None identified
**Caenorhabditis elegans**	**C48E7.6**	WS200	None identified
**Felis catus**	**ENSFCAG00000004349**	CAT	MVTQDPMLKAALPLFAR
**Gallus gallus**	**ENSGALG00000005426**	WASHUC2	MVNQEHMLKTTLPLFAR
**Pan troglodytes**	**ENSPTRG00000020782**	CHIMP2.1	MVTQESMLKAALPLFTR
**Ciona intestinalis**	**ENSCING00000013358**	JGI 2	None identified
	**ENSCING00000007527**	JGI 2	None identified
	**ENSCING00000004972**	JGI 2	None identified
**Bos taurus**	**ENSBTAT00000026827**	Btau_4.0	MVTQESMLKAALPLFAR
**Canis familiaris**	**ENSCAFG00000001537**	CanFam 2.0	LPLFAR
**Tursiops truncatus**	**ENSTTRG00000008037**	turTru1	MVTQESMLKATLPLFAR
**Loxodonta africana**	**ENSLAFG00000008954**	Loxafr3.0	MVKQESMLKAALPLFVR
**Takifugu rubripes**	**ENSTRUG00000006301**	FUGU 4.0	MHLSAFGYLFR**
**Gorilla gorilla**	**ENSGGOG00000014708**	gorGor3	MVTQESMLKAALPLFTR
**Cavia porcellus**	**ENSCPOG00000011267**	cavPor3	MVTQESMLKAALPLFAR
**Erinaceus europaeus**	**ENSEEUG00000001483**	eriEur1	MVTQESMLKAALPLFAR
**Homo sapiens**	**ENSG00000164946**	GRCh37	MVTQESMLKAALPLFTR
**Equus caballus**	**ENSECAG00000015567**	Equ Cab2	MVTQESMLKAALPLFAR
**Procavia capensis**	**ENSPCAG00000015286**	proCap1	MVKQESMLKAALPLFAR
**Dipodomys ordii**	**ENSDORG00000014455**	dipOrd1	LVTQESMLKAALPLFAR
**Echinops telfairi**	**ENSETEG00000015868**	TENREC	SMLKAALPLFVR
**Macaca mulatta**	**ENSMMUG00000008128**	MMUL1.0	MVTQESMLKAALPLFAR
**Callithrix jacchus**	**ENSCJAG00000012240**	Callithrix_Jacchus-3.2	MVTQESMLKAALPLFAR

**Oryzias latipes**	**ENSORLG00000020663**	HdrR	None identified
**Pteropus vampyrus**	**ENSPVAG00000014241**	pteVam1	MVTQESMLKAALPLFAR
**Myotis lucifugus**	**ENSMLUG00000016591**	myoLuc1	MVTQESMLKAALPLFAR
**Mus musculus**	**ENSMUSG00000059049**	NCBIM37	MGTQEPMLKAALPLFAR
**Microcebus murinus**	**ENSMICG00000015934**	micMur1	MVTQESMLKAALPLFAR
**Monodelphis domestica**	**ENSMODG00000014986**	monDom5	MVKQASMLKATLPLFAR
**Pongo pygmaeus**	**ENSPPYG00000019211**	ppYG2	MVTQESMLKAALPLFTR
**Sus scrofa**	**ENSSSCG00000005187**	Sscrofa9	No homologous exon Identified
**Ochotona princeps**	**ENSOPRG00000017066**	OchPri2.0	MVTQESMLKAALPLFAR
**Ornithorhynchus anatinus**	**ENSOANG00000022534**	Ornithorhynchus_anatinus-5.0	No homologous exon identidied
	**ENSOANG00000013004**	Ornithorhynchus_anatinus-5.0	No homologous exon identidied
	**ENSOANG00000010120**	Ornithorhynchus_anatinus-5.0	No homologous exon identidied
**Oryctolagus cuniculus**	**ENSOCUG00000010379**	oryCun2	MVTQESMLKAALPLFAR
**Rattus norvegicus**	**ENSRNOG00000022309**	RGSC 3.4	MGTQEPMLKTALPLFAR
**Sorex araneus**	**ENSSARG00000010446**	sorAra1	MVTHESMLKAALPLFAR
**Choloepus hoffmanni**	**ENSCHOG00000008350**	choHoff1	MVKQESMLKAALPLFAR
**Spermophilus tridecemlineatus**	**ENSSTOG00000003243**	speTri1	MVTQESMLKAALPLFAR
**Gasterosteus aculeatus**	**ENSGACG00000000932**	BROAD S1	None identified
**Tarsius syrichta**	**ENSTSYG00000004164**	TarSyr1	ARESMLKAPLPLFAR
**Tetraodon nigroviridis**	**ENSTNIG00000005289**	TETRAODON 8.0	None identified
**Tupaia belangeri**	**ENSTBEG00000013718**	tupBel1	MVTPEPMLKAALPLFAR
**Macropus eugenii**	**ENSMEUG00000012357**	Meug_1.0	MVKQASMLKATLPLFAR
**Xenopus tropicalis**	**ENSXETG00000003300**	JGI4.1	MGKQEPMLKAPLPLFAR
	**ENSXETG00000008875**	JGI4.1	None identified
**Taeniopygia guttata**	**ENSTGUG00000004583**	Taeniopygia_guttata-3.2.4	MVNREQMLKTALPLFAR
**Danio rerio**	**ENSDARG00000069473**	Zv7	None identified

Ensembl Frem1b orthologs

Organism	Ensembl Gene	Genome version	Sequence identified
**Anolis carolinensis**	**ENSACAG00000007607**	AnoCar1.0	None identified
**Ciona savignyi**	**ENSCSAVG00000001283**	CSAV 2.0	None identified
**Caenorhabditis elegans**	**C48E7.6**	WS200	None identified
**Ciona intestinalis**	**ENSCING00000013358**	JGI 2	None identified
	**ENSCING00000007527**	JGI 2	None identified
	**ENSCING00000004972**	JGI 2	None identified
**Takifugu rubripes**	**ENSTRUG00000000642**	FUGU 4.0	None identified
**Oryzias latipes**	**ENSORLG00000003500**	HdrR	None identified
**Gasterosteus aculeatus**	**ENSGACG00000003138**	BROAD S1	None identified
**Tetraodon nigroviridis**	**ENSTNIG00000002896**	TETRAODON 8.0	None identified
**Xenopus tropicalis**	**ENSXETG00000008875**	JGI4.1	None identified
**Danio rerio**	**ENSDARG00000062402**	Zv8	None identified
